# Tuning the Electronic Response of Metallic Graphene by Potassium Doping

**DOI:** 10.1021/acs.nanolett.2c03891

**Published:** 2022-12-23

**Authors:** Dario Marchiani, Andrea Tonelli, Carlo Mariani, Riccardo Frisenda, José Avila, Pavel Dudin, Samuel Jeong, Yoshikazu Ito, Francesco Saverio Magnani, Roberto Biagi, Valentina De Renzi, Maria Grazia Betti

**Affiliations:** † Physics Department, Sapienza University of Rome, Piazzale Aldo Moro 5, 00185Rome, Italy; ‡ Dipartimento di Scienze Fisiche, Informatiche e Matematiche (FIM), Università di Modena e Reggio Emilia, 41125Modena, Italy; § Synchrotron SOLEIL, Université Paris-Saclay, Saint Aubin, BP 48, 91192Gif sur Yvette, France; ∥ Institute of Applied Physics, Graduate School of Pure and Applied Sciences, University of Tsukuba, Tsukuba305-8573, Japan; ⊥ S3, Istituto Nanoscienze, Consiglio Nazionale delle Ricerche (CNR), Via Campi 213/A, 41125Modena, Italy

**Keywords:** nanoporous graphene, alkali metal doping, spectromicroscopy, plasmon

## Abstract

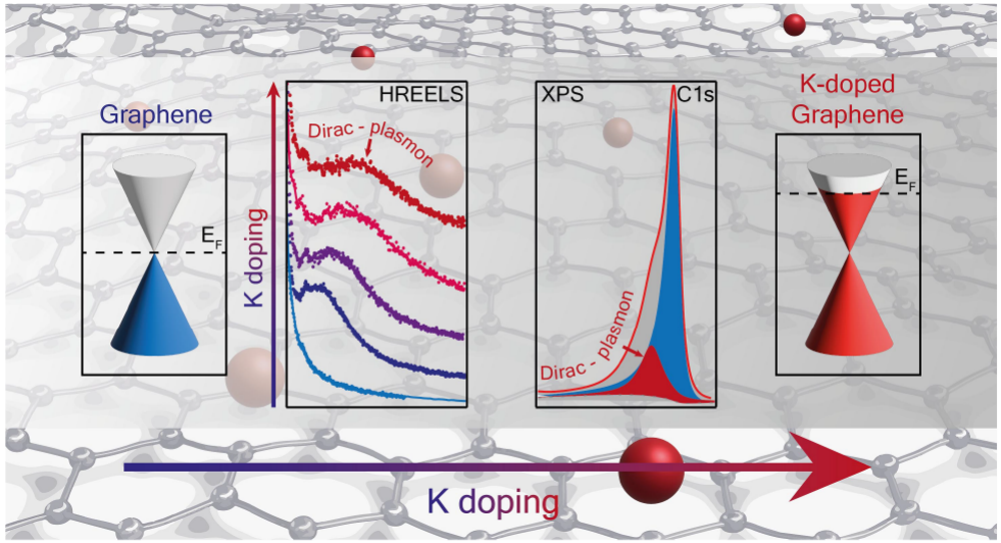

Electron doping of graphene has been extensively studied on graphene-supported surfaces, where the metallicity is influenced by the substrate. Herewith we propose potassium adsorption on free-standing nanoporous graphene, thus eluding any effect due to the substrate. We monitor the electron migration in the π* downward-shifted conduction band. In this rigid band shift, we correlate the spectral density of the π* state in the upper Dirac cone with the associated plasmon, blue-shifted with increasing K dose, as deduced by electron energy loss spectroscopy. These results are confirmed by the Dirac plasmon activated by the C 1s emitted electrons, thanks to spatially resolved photoemission. This crosscheck constitutes a reference on the correlation between the electronic π* states in the conduction band and the Dirac plasmon evolution upon in situ electron doping of fully free-standing graphene.

Semimetallic graphene (Gr) is characterized by the negligible density of state at the intrinsic Fermi level. An increase of electronic charge in the conduction band can significantly reduce the electrical resistivity, as observed also in graphite and carbon nanotubes.^1−3^ The Gr linear band dispersion gives rise to charge carriers with extremely high Fermi velocity,^4^ and a fine-tuning of the metallicity of Gr can not only affect the transport properties but also mediate ground states driven by a modified electron–phonon interaction^5−9^ and lead to exotic phenomena such as superconductivity.^10−12^

Alkali metals (AMs) can act as electron donors on carbon-based materials and organic systems,^2,13−24^ thanks to their simple electronic configurations, and the chemical doping can open new routes to explore the response of metallic Gr at increasing electron charge transfer, up to a downshift of the π* conduction band (CB) with the Fermi level by more than 1 eV into the CB.^2,21,22,25^ In particular, AM doping significantly affects the low-frequency dielectric response of graphene, which is determined by the collective charge oscillation of the π* electron density *n*, i.e., the so-called Dirac plasmon (DP). Doping can also be obtained in electrostatically gated graphene transistors, where the applied gate voltage moves the Fermi level up to a position of higher π* state density,^26,27^ slightly modifying the vibrational and plasmonic response coupling.^8^ However, this method cannot be effectively employed in free-standing Gr as proposed here because of the low efficiency of the gate and the structural instability of suspended Gr under an applied field.

Whereas theoretical calculations have often been carried out for AM on ideal Gr, experimental studies have been performed only for AM adatoms deposited on supported Gr, unraveling discordant conclusions, like a gap opening irrespective of the AM and the substrate^18,20^ or a straightforward Dirac cone shift, with unperturbed band topology.^21,22,25^ As frequently interpreted also for electron-doped graphite,^2^ a rigid band model shift induced by the charge migration can catch the salient phenomenology due to the AM doping in a low doping regime, where both the band topology and the high group velocity are preserved. As far as plasmon excitation is concerned, it has been extensively investigated with high-resolution electron energy loss spectroscopy (HREELS) in the case of substrate-supported^28−30^ graphene or by infrared/terahertz excitation light in a gated graphene,^31,32^ while to the best of our knowledge no detailed investigation has been yet reported for fully suspended graphene.

We herewith propose a fine control of the graphene metallicity, i.e., the doping level, giving insight into the interrelationship between the electronic spectral density in the conduction band and the plasmonic spectrum, by combining state of the art photoelectron spectromicroscopy and high-resolution electron energy loss spectroscopy. To circumvent the effects due to the substrate, we deposit potassium on fully unsupported large size and free-standing nanoporous graphene (NPG). NPG is a high-quality single- or double-turbostratic and low interacting layer graphene, with a continuous surface in space (see microscopy images in the Supporting Information, SI), with a negligible defect density that presents the hallmarks of an ideal semimetallic Gr layer, as recently shown by Raman and photoelectron spectroscopy studies.^33,34^ Thus, NPG can mimic an ideal graphene particularly apt for doping with AMs, monitoring the occupation of the π* conduction band correlated with the Dirac plasmon evolution.

A pivotal challenge to finely control the Fermi level position and the metallic state of Gr is to employ fully free-standing Gr specimens with high specific surface area, where alkali metals can homogeneously adsorb to form a thermodynamically stable arrangement. Nanoporous graphene is an ideal candidate for this purpose. It is constituted by a compact, bicontinuous interconnected three-dimensional (3D) arrangement of high-quality Gr veils, constituted by single or weakly interacting bilayer(s) of Gr, stacked in a twisted turbostratic arrangement, as recently assessed by transmission electron microscopy and Raman measurements.^33,34^ A scanning electron microscopy (SEM) image of this NPG is reported in Figure 1a (see also the SI).

**Figure 1:**
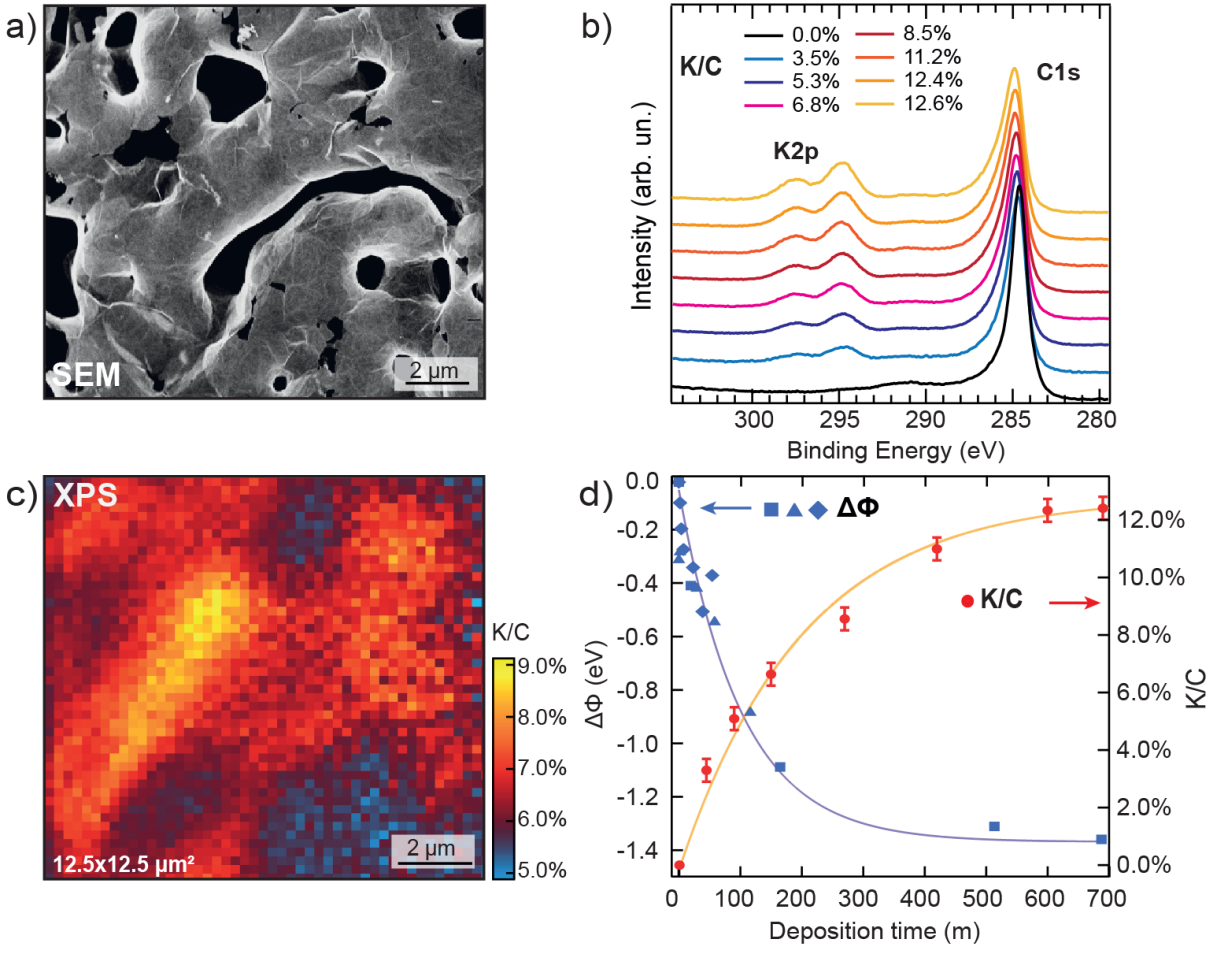
(a) SEM image of NPG (12.5 × 12.5 μm^2^). (b) C 1s and K 2p XPS core-level spectra of the K-NPG system as a function of potassium coverage. (c) Spatial mapping of the K 2p/C 1s intensity ratio in a 12.5 μm × 12.5 μm area with a 250 nm pixel step. (d) K 2p/C 1s intensity ratio (red symbols) and work function variation (ΔΦ, blue symbols, from different runs) as a function of K deposition time (continuous lines are only guidelines for the eye).

To determine the adsorption process and to quantify the potassium content on NPG during the K deposition in an ultrahigh vacuum, we employ X-ray photoelectron spectroscopy (XPS). The C 1s and K 2p core-level photoemission spectra for the clean NPG and for subsequent K exposures up to saturation coverage are displayed in Figure 1b. The K 2p core levels increase in intensity, preserving the same line shape, while the C 1s peak widens the tail toward higher binding energy (BE), as a function of K dose, as expected for a more metallic response, as will be discussed in more detail below. The micro-XPS C 1s and K 2p core-level spectra have been collected over a region of 12.5 μm × 12.5 μm, in steps of 250 nm, for an intermediate K coverage on Gr. The K:C at. % intensity ratio map, as obtained by the core-level area normalized to the photoionization cross sections,^35^ is reported in Figure 1c. The micro-XPS spatial mapping of the K:C normalized intensity ratio at the mesoscopic scale reveals a homogeneous and narrow distribution of the K adatoms (6.6 ± 0.7 at. %), as detailed in the SI. The average K:C core-level intensity ratio after normalization to each core-level excitation cross section^35^ is shown in Figure 1d as a function of K deposition time. It shows a saturation value of *I*_K_/*I*_C_ ≃ 0.12, compatible with the predicted (2 × 2) phase with a KC_8_ stoichiometry with each K adatom expected in the hollow sites at the center of one Gr honeycomb lattice every two lattices.^25^ This configuration is suggested by DFT-LDA predictions with an energy landscape of the stable (2 × 2) structure computed for K adsorbed on graphite,^2,17^ while potassium adsorbed on Gr supported on surfaces unravels a more complex phase diagram driven by thermodynamic forces and adsorption architectures, induced by the substrate.^20−22,25^

The work function (WF) variation with respect to the pristine NPG as a function of the K evaporation time is reported in Figure 1d. The WF (details on the measurement method in the Experimental Section) suddenly decreases and saturates at about −1.3 eV below the 4.7 eV value of the pristine graphene. A simple picture of the AM adsorption on Gr describes the partial ionization of potassium adatoms, generating a polarization field, due to the mutual repulsion of K adatoms leading to regular patterns, where electrons are donated to Gr without disturbing/warping the sp^2^ planar configuration.

A fine control of the evolution of the electronic structure close to the Fermi level as a function of K doping is reported in the UV photoemission valence band data shown in Figure 2a. The VB electron spectral density displays a downward rigid shift of the π states as a function of K dose. Correspondingly, an increased CB density of states appears at the Fermi level, as clearly unveiled by the zoomed in low binding energy region reported in Figure 2b. It is generally accepted that alkali metals donate electrons to Gr, preserving the band topology and the group velocity in a low doping regime, resulting in a rather rigid shift of the Gr π and π* bands. The charge carrier density and the corresponding doping level, i.e., the number of extra electrons per C atom, can be directly related to the energy shift of the Dirac point *E*_D_ with respect to the Fermi level, i.e., (*E*_F_ – *E*_D_) = Δ*E*, as can be verified in panel (c) of Figure 2 (details in the SI).

**Figure 2:**
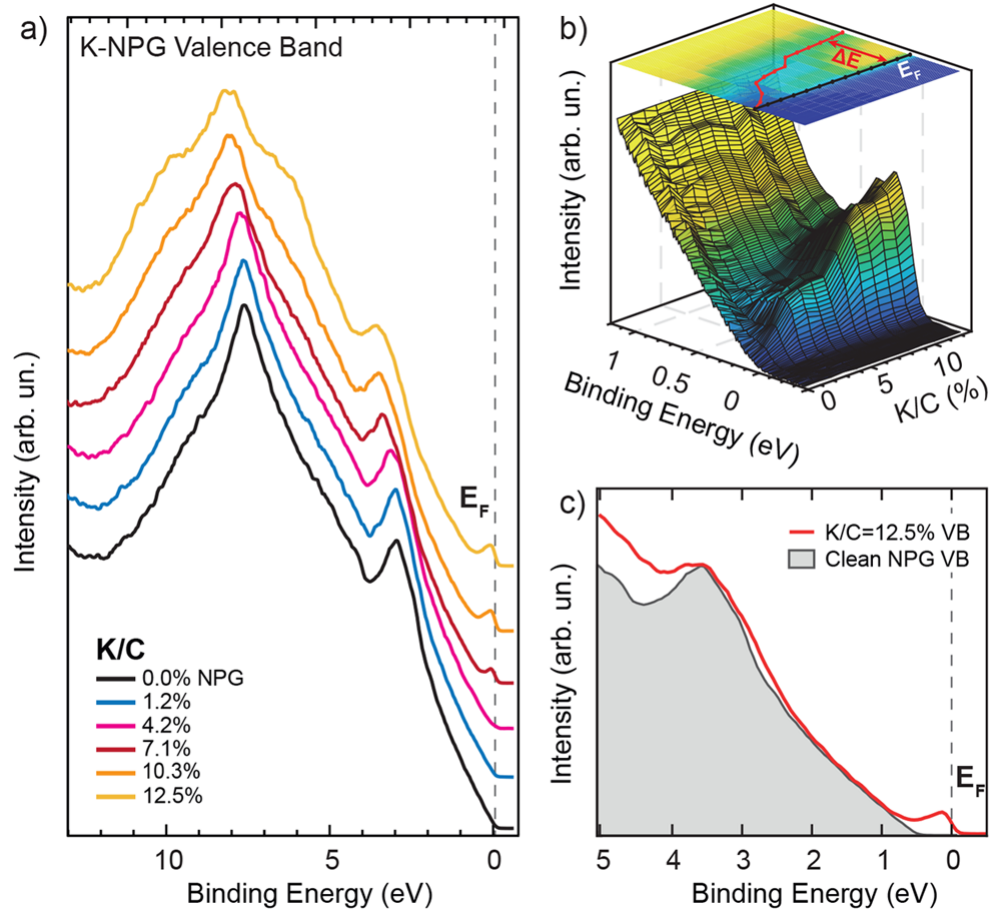
(a) UV photoemission spectral density of the K-NPG system as a function of K exposure (spectra vertically stacked for the sake of clarity). (b) Perspective 3D view of the spectral density evolution of the VB close to the Fermi level (black line) as a function of K dose; the Dirac point energy shift Δ*E* due to charge injection is reported as a red line in the top projection of the 3D perspective view. (c) Experimental spectral density of K-NPG at saturation coverage (red line) compared with clean NPG, shifted by −0.6 eV in spectral density (gray filled area).

From the VB shift, we can therefore approximately calculate the injected charge density according to the formula:  Taking into consideration the high K doping, we introduce a renormalized Fermi velocity by a factor ∼0.8^36^ with respect to that of pristine free-standing graphene (*v*_F_ = 1 × 10^6^ m/s), and the estimated accumulated charge is *n* = (3.4 ± 0.8) × 10^13^ cm^–2^ for the saturation coverage.

The electron chemical doping of free-standing Gr by K adsorption provides the opportunity to investigate the collective charge oscillations of the electron density in the upper Dirac cone. Combining HREEL data with complementary UPS measurements, we are able to follow the evolution on the plasmon excitation as a function of K doping.

A selected set of HREELS spectra of K-NPG are reported in Figure 3, as a function of the K-induced Dirac cone energy shift Δ*E* (as determined by UV photoemission and checked by XPS). In panel (a) the spectrum of the pristine NPG is compared with that corresponding to Δ*E* = 0.28 ± 0.02 eV. While the former is essentially featureless, displaying a Drude-like quasi-elastic tail characterized by a power-law line shape, upon K doping we observe the growth of a clearly defined and asymmetric feature, related to the plasmon excitation. Figure 3b reports the evolution of the observed plasmon feature—obtained after proper subtraction of the power-law background (procedure detailed in the SI)—for increasing values of Δ*E* and the Dirac cone energy shift. The loss feature is quite asymmetric, showing a blue-shift and a marked broadening upon increasing potassium doping. The energy value associated with the plasmon mode—corresponding to the loss feature maximum—is reported in the inset of Figure 3b, as a function of the K-induced Dirac cone energy shift. The plasmon blue-shift can be associated with the increased occupation of the lowest π* band of Gr by electron charge donated by potassium, in agreement with previous Gr-doped experimental results.^30,37^ We observe a very notable increase in plasmon broadening vs K doping, corresponding to an increased damping due to an increased probability of e-h π–π* pair excitations.^38^ The nature of the band asymmetry and the possible presence of more complex multiple modes^28,39^ are controversial and still debated.

**Figure 3:**
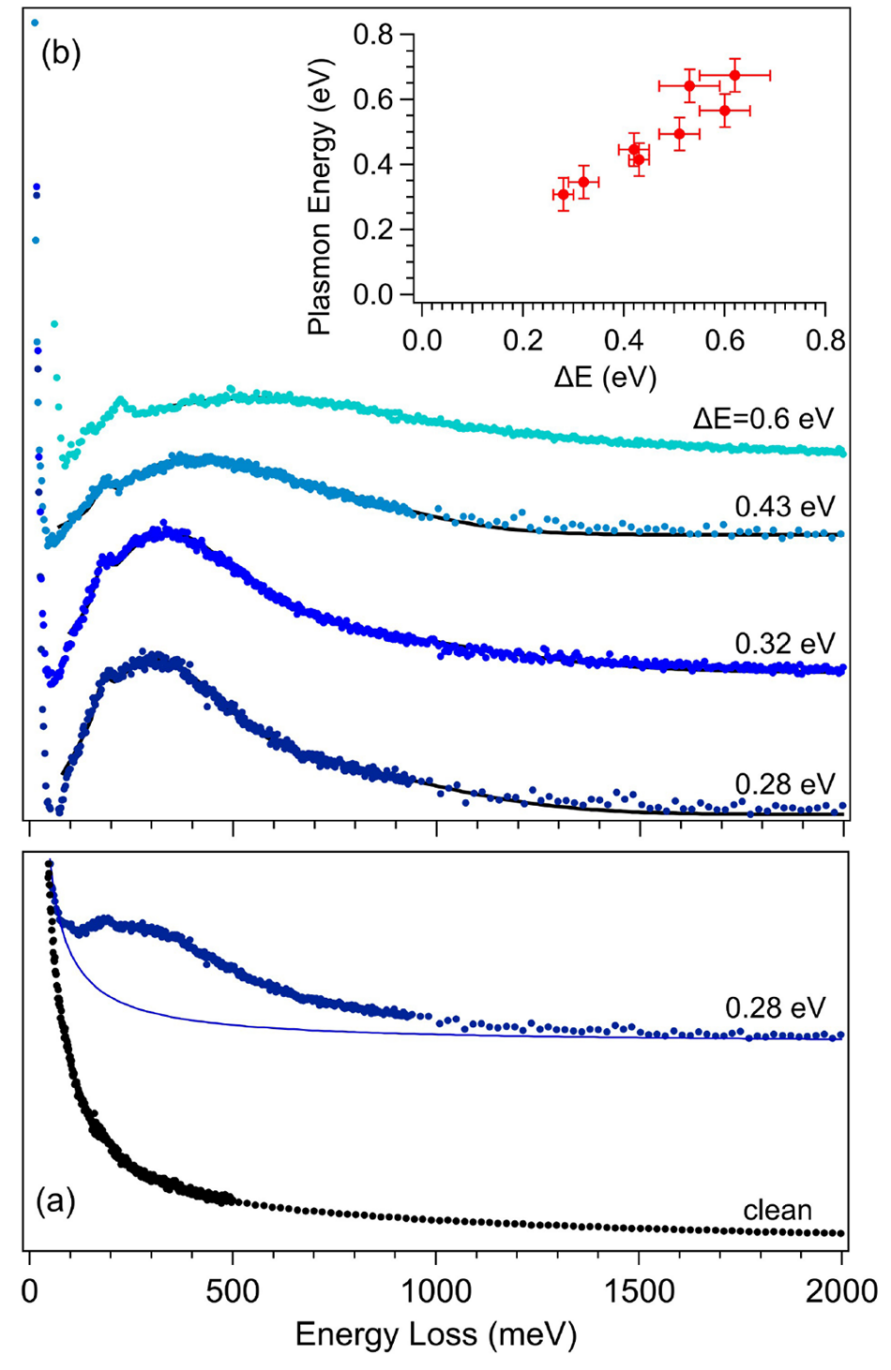
(a) HREEL spectra of the pristine (black symbols) and doped NPG (blue symbols) for Δ*E* = 0.3 eV, showing the growth of the doping-induced plasmon feature. A small and narrow peak at 179 meV is also observed and attributed to residual contaminants (CH bending vibration). (b) Evolution of the plasmon feature as a function of K-induced Dirac cone-energy shift with respect to Δ*E*, as determined from UPS data. The plasmon loss feature is obtained from the raw HREEL spectra after subtraction of a power-law background (shown as a thin solid line in (a)), as described in the SI. In the inset, the energy of the plasmon feature is reported as a function of the Dirac-cone energy shift with respect to *E*_F_ (Δ*E*).

Interestingly, the here reported evidence of a doping-dependent plasmonic feature helps to clarify the modification of the C 1s line shape upon K adsorption, as shown in Figure 1b. The C 1s core-level line shape changes as a function of K dose, becoming broader and more asymmetric toward higher binding energy. A deeper insight into this issue can be unravelled by a careful line shape analysis of the micro-XPS C 1s core level taken at high energy resolution over a region of 7.5 μm × 7.5 μm. The spatially resolved K:C normalized intensity ratio reported in the inset to Figure 4a shows areas with lower or higher K content (albeit with a rather narrow distribution, see SI). We pick up two exemplary C 1s peaks with limiting K:C ratios, reported in panel (a) of Figure 4. Beyond the peak asymmetry, a prominent and well-resolved shoulder is resolved at higher binding energy with respect to the main C 1s peak (sp^2^ component), whose intensity is proportional to the local coverage of K.

**Figure 4:**
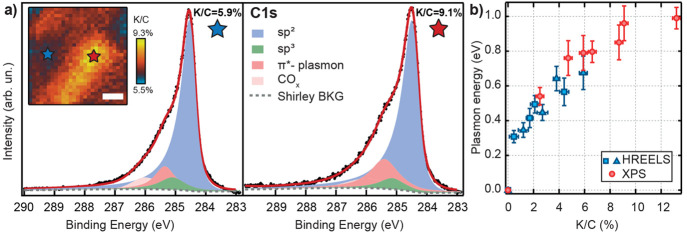
(a) Micro-XPS spectra with high energy resolution of the C 1s core level taken in two different spatial regions, with lower (left panel; blue star) and higher (right panel; red star) K coverage; experimental data (black dots), total fitting curve (red line), and sp^2^ (blue area) and sp^3^ (green area) components. A prominent shoulder, due to electron charge donation and ascribable to a π*-plasmon excitation, is evident at high BE (0.6–0.8 eV from the main sp^2^ component, pink area) as a function of the K coverage. Inset: spatially resolved micro-XPS map of the C 1s core-level intensity: 7.5 × 7.5 μm^2^ image formed by 250 × 250 nm^2^ pixels. (b) Energy evolution of the maximum intensity of the π*-plasmon component obtained from the XPS fitting deconvolution (red circles) and of the energy of the maximum of HREELS π*-plasmon (light blue symbols), as a function of the K:C concentration.

It is instructive to have a closer look at the fitting components of the C 1s core level (details in the SI). In fact, the high-BE spectral tail cannot be merely explained by an increasing metallic asymmetry.^40^ The presence of an additional high-BE feature is essential to reproduce the experimental results, which can be ascribed to the excitation of the Dirac plasmon by the outgoing photoelectrons. The plasmon energy increases as a function of K doping (Figure 4b) due to the increasing charge density in the occupied portion of the π* band.

Only few experimental papers have observed an unresolved shoulder on the high-BE side of the main C 1s peak in AM-doped supported Gr and graphite,^17,41^ but the interpretation of its origin was controversial. The asymmetric broadening of the C 1s core level upon doping has been attributed to an increased density of states at the Fermi level and/or to the formation of electron–hole pairs fostered by an increased phase space for these intraband/interband transitions with doping level. It was attributed to shakeup satellite loss processes enabled by the formation of the metallic adsorbate layer (for example, in K–graphite^17^), but no experimental investigation of the C 1s core level of AM-doped Gr has been taken into consideration the occurrence of an effective and distinct plasmon loss. From the theoretical point of view, Despoja et al.^42^ simulated the C 1s core level of free-standing graphene for different levels of doping, enlightening the increasing probability of interband transitions from π to π* bands as a function of doping, but also claimed the occurrence of a π* plasmon due to the charge migration in the upper Dirac cone, in agreement with our findings.

A key finding of our high energy resolved micro-XPS experiment is that the C 1s core-level high-BE side can be interpreted as the superposition of the electron–hole pairs due to the interband transition from π to π* bands, giving rise to the asymmetric tail of the main peak (see also SI), and the distinctive well-resolved π* plasmon peak due to the electron fluctuations in the conduction band, while the contamination gives only a negligible residual (see SI). It is worth noting that a rigid band model can explain the evolution of the plasmon as a function of electron doping if the Dirac cone Δ*E* is in an energy range comparable with the van Hove singularity.^41,42^ The quasiparticle energy as a function of K dose can be compared to the maximum of the plasmonic feature measured by HREELS. In Figure 4b, we report the π*-plasmon energy as evaluated, collecting all the experimental data (HREELS and XPS), as a function of K dose. We observe a clear plasmon energy dependence vs K:C content, proportional to the charge density in the upper Dirac cone.

In conclusion, electron doping of graphene has been obtained by potassium deposition onto fully free-standing nanoporous graphene. The parallel analysis of the electron spectral density near the Fermi level and of the Dirac plasmon evolution clearly unveils a uniform in situ electron doping of fully free-standing and unsupported graphene. Charge donation causes the partial occupation of the π* upper Dirac band, without significantly affecting the spectral density distribution of the Dirac cone electronic states except for an energy downward shift, as determined by UV photoemission, in agreement with a rigid band model. HREELS has been successfully employed to fingerprint the correlation between the plasmonic excitations due to the charge migration in the conduction band and the Dirac cone shift measured by photoemission. Moreover, we further confirm the dependence of the plasmonic modes on the K dose by means of the extrinsic plasmon component in the core-level photoemission. Combining HREELS and UPS data, we investigate the (free-standing) graphene plasmonic excitation as a function of the K doping. These correlated experimental findings on the plasmonic response and Dirac cone evolution in electron-doped free-standing graphene establish a reference point, though a careful theoretical investigation can distinctively clarify the evolution of the quasi-particle spectrum in the phase space.

## Experimental Methods

### Sample Preparation

Nanoporous graphene was grown through a nanoporous Ni template by means of chemical vapor deposition (CVD). Ingots of Ni_30_Mn_70_ alloy were first synthesized by melting pure Ni and Mn in an Ar-protected arc melting furnace, and then they were annealed to become microstructured and composition-homogeneous alloys and rolled to thin films. In order to obtain the nanoporous Ni template, the NiMn alloy sheet was chemically dealloyed with 0.5 M ammonium sulfate for nanoporous Ni. The nanoporous Ni was used as a CVD substrate, and benzene was then used as precursor for CVD graphene growth at 900 °C for 5 min. The graphene sheet covering nanoporous Ni and presenting its same three-dimensional morphology was subsequently exfoliated by chemical dissolution of the Ni template by 1.0 M hydrochloric acid. The synthesis and preparation process are described in detail elsewhere.^43−48^

Prior to the spectroscopic data acquisition in each UHV apparatus in each laboratory, the NPG samples were degassed at 600–620 °C for several hours to remove contaminants from air exposure.^49^

Potassium was sublimated in UHV (in the 10^–10^ mbar range) by using commercial SAES getter dispensers, after overnight degassing at currents slightly below the sublimation onset. The sublimation rate was kept as constant as possible in each measurement set, and it was similar in the different UHV chambers, by using equivalent dispensers mounted in each apparatus.

### Photoelectron Spectroscopy

The spectromicroscopy photoemission experiments were performed at the ANTARES beamline of the SOLEIL synchrotron radiation facility (France). The nano-X-ray photoelectron spectroscopy (XPS) microscope is equipped with two Fresnel zone plates for beam focusing, ensuring spatial resolution in raster imaging down to the 400–500 nm scale. The NPG sample was positioned at the common focus point of the hemispherical analyzer and the Fresnel zone plates, by mounting it onto a precision positioning stage. In the imaging mode the spectrum of photoelectrons in the desired energy range was collected on the 2D detector at each point of the spatial raster having a submicrometer step. Core-level spectra were taken with 350 eV photon energy, to enhance the surface sensitivity, and the analyzer pass energy was set to 50 and 200 eV for the spatially integrated and resolved modes, respectively. Measurements were taken under UHV (10^–10^ mbar), and the sample was kept cooled at a liquid nitrogen temperature during measurements, to avoid radiation damage.^34^

The UPS valence band data have been taken at the Lotus laboratory (Sapienza University, Roma) by using a Gammadata VUV 5000 microwave excited and monochromatized He source, with He*II*_α_ radiation (40.814 eV). Photoelectrons were analyzed with an electrostatic hemisferical Scienta SES 200 analyzer equipped with a multichannel plate detector, operated with an overall 20 meV energy resolution, analogous to the setup employed at the ANTARES beamline. The work function has been determined through the measurement of the whole spectral width from the secondary electron cutoff to the Fermi level, after biasing the sample by −3 V with respect to ground and using He*I*_α_ radiation (21.218 eV).

### High-Resolution Electron Energy Loss Spectroscopy

HREELS measurements were performed at the SESAMO laboratory (University of Modena) with a LK 5000 spectrometer (3.5 meV resolution), with primary beam energy of 9 eV and incident angle θ_*i*_ = 52°. We remark that the NPG three-dimensional (3D) morphology is characterized by a rather broad distribution of surface orientations, thus hindering a precise determination of the exchanged momentum *q*. Complementary XPS and UPS measurements were also performed, to combine information on coverage and Fermi energy shift, respectively. XPS core-level spectra were taken using a nonmonochromatic X-ray source (Al Kα 1486.7 eV) and acquired with an Omicron EA-125 hemispherical analyzer. The UPS valence band data were taken using He*I*_α_ radiation with the same hemispherical analyzer.

## Supporting Information Available

Transmission electron microscopy images of this NPG sample, experimental procedures for the HREELS background subtraction and spectral analysis, and details on the valence band alignment and C 1s fitting procedure (PDF)
