# Supplementary material for: Tuning the Electronic Response of Metallic Graphene by Potassium Doping
Source: Nano Lett. 2022 Dec 23;23(1):170–6. doi: 10.1021/acs.nanolett.2c03891 (PMC9838101; doi:10.1021/acs.nanolett.2c03891)
Supplement: Supplementary file 1 — nl2c03891_si_001.pdf [file nl2c03891_si_001.pdf]

Supporting Information: Tuning the electronic response of metallic graphene by potassium doping

Dario Marchiani,[†] Andrea Tonelli,[‡] Carlo Mariani,[†] Riccardo Frisenda,[†] José Avila,[¶] Pavel Dudin,[¶] Samuel Jeong,[§] Yoshikazu Ito,[§] Francesco Saverio Magnani,[‡] Roberto Biagi,[‡,‖] Valentina De Renzi,[*,‡,‖] and Maria Grazia Betti[*,†]

†*Physics Department, Sapienza University of Rome, Piazzale Aldo Moro 5, 00185 Rome (Italy)*

‡*Dipartimento di Scienze Fisiche, Informatiche e Matematiche (FIM), Università di Modena e Reggio Emilia, 41125 Modena (Italy)*

¶*Synchrotron SOLEIL, Université Paris-Saclay, Saint Aubin, BP 48, 91192 Gif sur Yvette (France)*

§*Institute of Applied Physics, Graduate School of Pure and Applied Sciences, University of Tsukuba, Tsukuba, 305-8573 (Japan)*

‖*S3, Istituto Nanoscienze, Consiglio Nazionale delle Ricerche (CNR), Via Campi 213/A, 41125 Modena (Italy)*

E-mail: vderenzi@unimore.it; mariagrazia.betti@uniroma1.it

Microscopy images of the NPG sample

A selection of Transmission Electron Microscopy (TEM) images of the NPG sample is reported in Fig. S1. NPG mostly presents one and two-layers graphene veils, as widely

discussed in previous works.[1,2] In particular, here we show regions of bi-layer NPG at different spatial resolutions (left, lower side 5 nm); zones with different turbostratic orientations (center and right panels), where different moiré modulations are visible. This structural modulation of the two-layers is characteristic of a non-Bernal stacking, thus with a turbostratic and less interacting behavior.

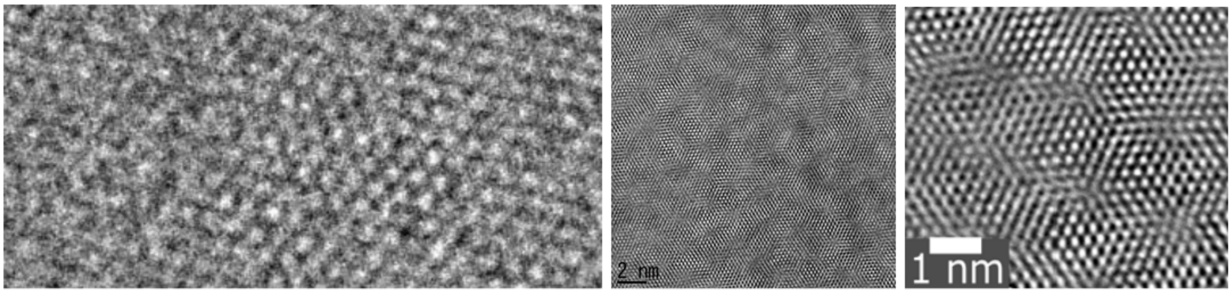

Figure S1: TEM images of bi-layer NPG at different spatial scales; (left) lower side 5 nm; (center and right) NPG zones with turbostratic two-sheet graphene and different moiré patterns. Detailed discussion in ref.[2]

Homogeneity of K deposition on NPG: XPS spectromicroscopy data

To quantify the degree of homogeneity in the K distribution on NPG, the C 1s and K 2p core levels have been measured over a region of 12.5μm$\times$12.5μm, sampled in steps of 250 nm. For each point, we have performed a core/level fitting of each core level and the K:C at. % intensity ratio, normalized to their respective photoionization cross sections,[3] are reported in the spectromicroscopy map of Fig. 1c in the main paper.

To better evaluate the homogeneity, the intensity ratio histogram of the occurrences as derived from the mapping are displayed in the histogram of Fig. S2. The distribution, centered at the value of about 6.6 at. % presents a width of $\pm$0.7 at. %, thus suggesting a rather homogeneous and narrow spatial distribution of the K atoms on the Gr mesh.

Worth to notice that the narrow distribution of K concentration on NPG is independent

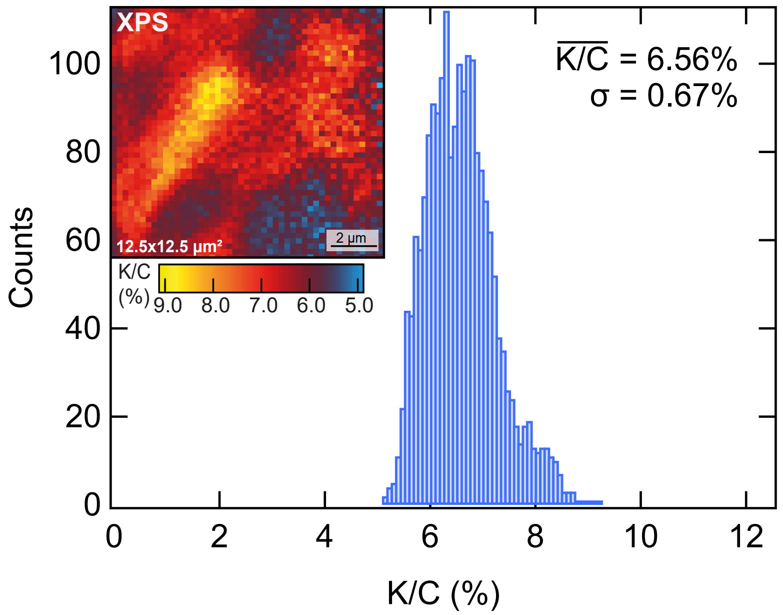

Figure S2: Histogram of the occurrence of the K:C at. % intensity ratio in a 12.5μm$\times$12.5μm area of the K-NPG sample, as deduced from the XPS spectromicroscopy data. Inset: XPS spectromicroscopy map of the normalized K:C core level intensity ratio.

on the sampled area, for example the equivalent K:C histogram distribution results as 6.8$\pm$0.7 at. % in the zone shown in the inset to Fig. 4 of the main paper.

Valence Band data analysis

Valence band data of K-doped graphene has been analyzed by comparing the spectral density evolution with the clean undoped NPG reference. In particular, within the approximation of a rigid band model, the signal of the doped samples has been shifted with respect to the clean one by a ΔE value (see Fig. 2 of the main paper), as in the following. We aligned the 2p-$\pi-$peak around 3 eV BE and the decreasing linear slope of the undoped graphene with the same features of the K-NPG for each K deposition level. As a result, the clean NPG spectrum has been shifted towards higher binding energy values and the doping degree was determinate by this total shift, as shown in Fig. 2 c) of the main text (ΔE=-0.6 eV). Comparing the clean NPG spectrum with the doped one is consistent with the rigid band

model, since no significant distortions of the Valence band results as a function of the doping level, allowing to determine the doping degree by the shift of the bands.

HREELS spectral data analysis

EELS background subtraction

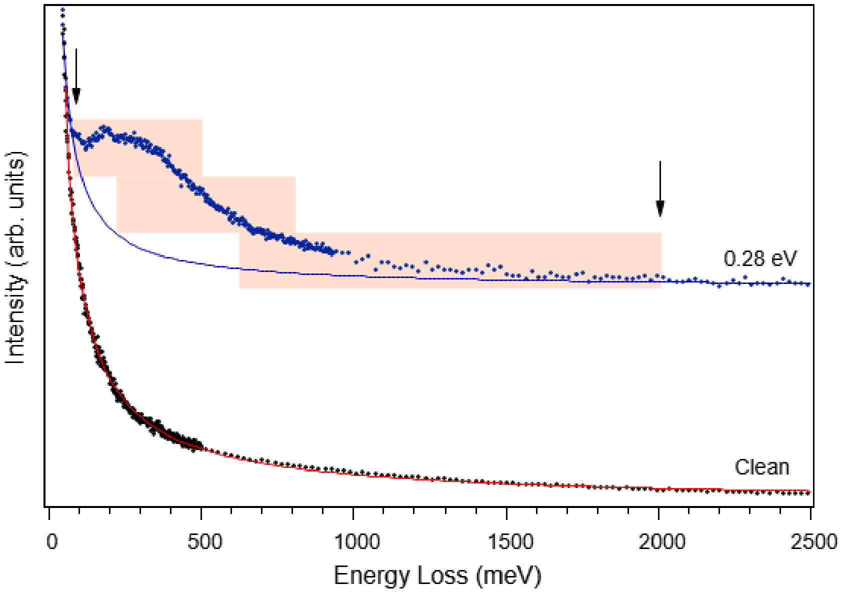

Figure S3: HREEL spectra of the pristine (black symbols) and doped NPG (blue symbols) for ΔE=0.3 eV, together with their corresponding fitting curves. In order to determine the background of the doped-NPG curve, the region of the plasmon loss (indicated by arrows and by the shaded area) has been masked and therefore not taken into account in the fitting procedure.

In Fig. S3, the EEL spectra of the pristine NPG sample is reported (black symbols), along with its power-law fitting-curve (red continuous line), attributed to the Drude-like metallic tail. The blue-symbol curve illustrates how K-doping induced the growth of the plasmon feature on top of the continuum-loss tail. In this case, the background is determined as a power-law, shown as a continuum line. The fitting curve is obtained by masking the portion of the spectrum where the plasmon loss is observed (shaded region) - thus taking only into account the quasi-elastic tail and the constant background in the low- energy and high-energy

regions of the spectrum, respectively. A similar procedure was followed for all other curves shown in Fig. 3 of the main paper, corresponding to increasing values of the K-doping.

Plasmon lineshape

The plasmon lineshape obtained after background subtraction is characterised by a distinct asymmetry. A similar finding has been reported in the case of supported epitaxial graphene on both [SiC and Ir(111) substrates] and accounted for considering two plasmonic features, i.e. the standard and a multipole plasmon.[4] Moreover, recent theoretical studies predicted the occurrence of two distinct plasmonic modes in extrinsic graphene, referred to ad Acoustic and Dirac plasmon,[5,6] although with discordant expected intensity among the modes. It is here relevant to notice that in our case, due to the 3D nature of the NPG sample surface, the NPG EELS spectra results from partial integration over different values of the exchanged momentum q. This fact, together with the predicted q-dispersion of the plasmon energy, may also explain the plasmon-loss asymmetric feature. A more throughout investigation on the origin of this lineshape would require a single-domain, well-oriented and still free–standing specimen and is therefore beyond the scopes of the present work.

C 1s core level fitting procedure

The experimental C 1s core level data have been fitted with a number of components associated to the different bonding environment, after subtracting a Shirley background. We mainly used Voigt lineshape peaks, i.e. Lorentzian–Gaussian curves taking into account both the intrinsic linewidth (Lorentzian) and the overall experimental uncertainty (Gaussian). The main component, represented by the sp^2 peak, has been fitted with an asymmetric Doniach–Sunjic lineshape, whose asymmetry parameter is ≤ 0.1 for the pristine clean NPG[7] and is due to the semimetallic nature of Gr.

Referring to the C 1s data and fitting curves reported in Fig. 4 of the main paper

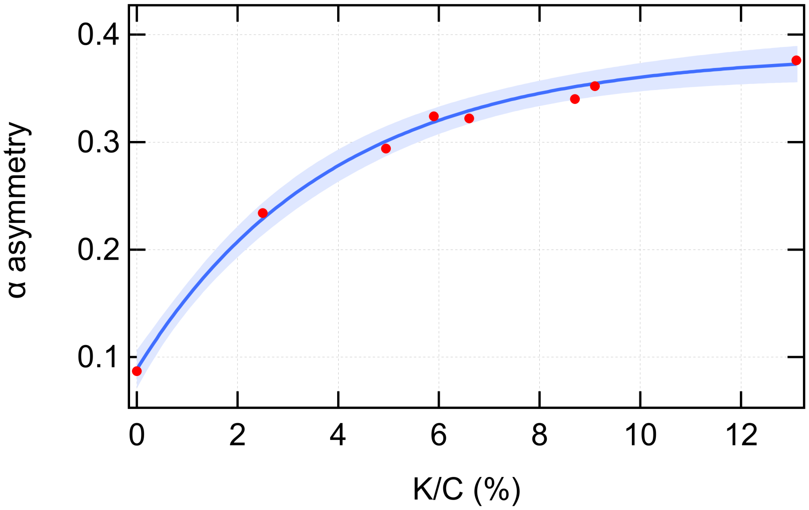

Figure S4: Variation of the asymmetry parameter of the sp^2 component, as a function of K deposition (K/C concentration in percentage). Red dots: experimental data, blue line: eyes guide-line, light-blue region: 68% confidence region.

representing K-doped NPG, we can observe the main sp^2 peak at 284.5 eV BE, a small sp^3 peak at 285.1 eV due to the slight bond deformation at wrinkled/bent regions always present in nanoporous Gr,[2,8] a very small (less than 5%) residual C-to-O structure at 286.1 eV,[9] and the intense plasmon excitation at 285.4 eV, with a width of about 0.8 eV, associated to the charge density in the π^* band, whose energy variation as a function of doping is reported in panel b) of Fig. 4 in the main paper.

Apart from the presence of this evident plasmon peak widely discussed in the main paper, the extrinsic intra/interband π-π^* transitions excited during the photoemission process are reflected into the asymmetry of the main sp^2 component. The fitting asymmetry parameter as a function of K doping is shown in Fig. S4. The asymmetry parameter increases from 0.09 (as expected in pristine graphene[7]) to about 0.37 at K saturation coverage, indicating an increased metallicity of the system.

References

(1) Ito, Y.; Tanabe, Y.; Qiu, H.-J.; Sugawara, K.; Heguri, S.; Tu, N. H.; et al., High-Quality Three-Dimensional Nanoporous Graphene. *Angewandte Chemie International Edition* **2014**, *53*, 4822–4826.

(2) Di Bernardo, I.; Avvisati, G.; Mariani, C.; Motta, N.; Chen, C.; Avila, J.; Asensio, M. C.; Lupi, S.; Ito, Y.; Chen, M.; Fujita, T.; Betti, M. G. Two-Dimensional Hallmark of Highly Interconnected Three-Dimensional Nanoporous Graphene. *ACS Omega* **2017**, *2*, 3691–3697.

(3) Yeh, J.; Lindau, I. Atomic subshell photoionization cross sections and asymmetry parameters: 1≤ Z≤ 103. *Atomic data and nuclear data tables* **1985**, *32*, 1–155.

(4) Pfnür, H.; Langer, T.; Baringhaus, J.; Tegenkamp, C. Multiple plasmon excitations in adsorbed two-dimensional systems. *Journal of Physics: Condensed Matter* **2011**, *23*, 112204.

(5) Pisarra, M.; Sindona, A.; Riccardi, P.; Silkin, V. M.; Pitarke, J. M. Acoustic plasmons in extrinsic free-standing graphene. *New Journal of Physics* **2014**, *16*, 083003.

(6) Despoja, V.; Novko, D.; Lončarić, I.; Golenić, N.; Marušić, L.; Silkin, V. M. Strong acoustic plasmons in chemically doped graphene induced by a nearby metal surface. *Phys. Rev. B* **2019**, *100*, 195401.

(7) Lacovig, P.; Pozzo, M.; Alfe, D.; Vilmercati, P.; Baraldi, A.; Lizzit, S. Growth of dome-shaped carbon nanoislands on Ir (111): the intermediate between carbidic clusters and quasi-free-standing graphene. *Physical Review Letters* **2009**, *103*, 166101.

(8) Di Bernardo, I.; Avvisati, G.; Chen, C.; Avila, J.; Asensio, M. C.; Hu, K.; Ito, Y.; Hines, P.; Lipton-Duffin, J.; Rintoul, L.; Motta, N.; Mariani, C.; Betti, M. G. Topology

and doping effects in three-dimensional nanoporous graphene. *Carbon* **2018**, *131*, 258 – 265.

(9) Jiménez-Arévalo, N.; Leardini, F.; Ferrer, I. J.; Ares, J. R.; Sánchez, C.; Saad Abdelnabi, M. M.; Betti, M. G.; Mariani, C. Ultrathin Transparent B–C–N Layers Grown on Titanium Substrates with Excellent Electrocatalytic Activity for the Oxygen Evolution Reaction. *ACS Applied Energy Materials* **2020**, *3*, 1922–1932.
